# Deciphering the Gene Expression and Alternative Splicing Basis of Muscle Development Through Interpretable Machine Learning Models

**DOI:** 10.3390/biology14081059

**Published:** 2025-08-15

**Authors:** Xiaodong Tan, Minjie Huang, Yuting Jin, Jiahua Li, Jie Dong, Deqian Wang

**Affiliations:** 1 Institute of Animal Husbandry and Veterinary Science, Zhejiang Academy of Agricultural Sciences, Hangzhou 310021, China; tanxd@zaas.ac.cn (X.T.);; 2 Key Laboratory of Livestock and Poultry Resources (Poultry) Evaluation and Utilization, Ministry of Agriculture and Rural Affairs, Hangzhou 310021, China

**Keywords:** alternative splicing, breast muscle, chicken, machine learning, shapley additive exPlanations

## Abstract

In chickens, meat yield is a crucial trait in breeding programs. Identifying key molecular markers associated with increased muscle yield is essential for breeding strategies. This study applied transcriptome sequencing and machine learning methods to examine gene expression and alternative splicing (AS) events in muscle tissues of commercial broilers and local chickens. On the basis of differentially expressed genes (DEGs) and differentially spliced transcripts (DSTs) significantly related to breast muscle weight percentage (BrP), high-accuracy prediction models were developed by evaluating 10 machine learning models (e.g., eXtreme Gradient Boosting (XGBoost), Generalized Linear Model Network (Glmnet)). Feature importance was assessed using the Shapley Additive exPlanations (SHAP) method. The results revealed that 50 DEGs and 95 DSTs contributed significantly to BrP prediction. The XGBoost model achieved over 90% accuracy when using DEGs, and the Glmnet model reached 95% accuracy when using DSTs. Through Shapley evaluation, genes and AS events (e.g., ENSGALG00010012060, HINTW, and VIPR2-201) were identified as having the highest contributions to BrP prediction. Additionally, the breed effect was effectively mitigated. This study introduces new candidate genes and AS targets for the molecular breeding of poultry breast muscle traits, offering a paradigm shift from traditional gene mining approaches to artificial intelligence-driven predictive methods.

## Simple Summary

Chicken is the most consumed meat globally and represents the most cost-effective protein source. Enhancing chicken production is crucial for ensuring food supply and security. While large-scale expansion of farming significantly increases environmental burdens, selective breeding employing molecular biology and genetics is the most efficient and vital approach. In this study, we investigated local chicken breeds (Xianju chicken) and commercial broilers. We identified genes and regulatory mechanisms differentiating high and low breast muscle weight. Combining these findings with machine learning methods revealed that these genes exhibit high accuracy in predicting meat production performance. Our findings provide important targets for chicken genomic selection and design breeding, enabling the improvement of chicken meat production efficiency through specific combinations.

## 1. Introduction

With the continuous growth of the global population and improvements in living standards, the demand for meat has increased significantly. Among various meat sources, chicken is particularly popular because of its high protein content, low fat content, and relatively low production costs. In 2019, chicken became the most consumed meat worldwide, accounting for more than 35% of total meat consumption. Its production is expected to continue growing steadily in the coming years [1]. Compared with pork and beef, chicken offers several advantages, including a shorter production cycle and lower feed conversion ratios [2], making it a more efficient source of protein [3]. Over several decades of genetic selection, the meat production performance of chickens has improved significantly [4]. For example, at 8 weeks of age, the body weight of chickens can increase threefold, with breast muscle weight increasing by more than 80% and breast muscle proportion reaching more than 25% [4]. This significant progress relies on continuous genetic selection and the exploration of molecular markers (e.g., genes, variants, alternative splicing (AS)) related to breast muscle traits and the investigation of potential genetic mechanisms.

Examining genes associated with meat yield at the gene expression level is a common research approach. For example, Tan et al. identified SRY-Box transcription factor 6 (*SOX6*) as a key gene for balancing muscle yield and meat quality by comparing gene expression differences between high and low breast muscle phenotypes and conducting Mendelian randomization analysis [5]. Similarly, Zambonelli et al. analyzed gene expression patterns in heterogeneous and normal muscle tissues and identified 204 differentially expressed genes, e.g., Cysteine and glycine-rich protein 3 (*CSRP3*) [6]. While these studies highlight genes of interest, the regulatory mechanisms underlying differential expression patterns remain unclear. Research indicates that more than 90%, 80%, and 60% of genes in the genomes of humans [7], pigs [8], and chickens [9], respectively, are regulated by AS. Studies have shown that AS modifications play significant roles in muscle development by influencing mRNA stability, localization, or translation processes [10]. For example, isoforms of Myostatin (*MSTN*) in poultry can negatively regulate the processing of myostatin precursors in muscle cells, thereby blocking the *MSTN*-mediated inhibition of muscle development [11] Additionally, Chen et al. reported that two Transformer 2 beta homolog (*TRA2B*) isoforms modulate the TGF-β signaling cascade differently through AS of Transforming growth factor beta receptor 2 (*TGFBR2*), leading to distinct roles in myogenic differentiation [12]. Therefore, analyzing the AS patterns in muscle tissues can help enhance our understanding of the regulatory mechanisms governing meat yield.

Compared with traditional correlation analysis and differential expression gene analysis, recent machine learning (ML) methods can efficiently fit complex data and achieve higher predictive accuracy while also providing better interpretability for nonlinear causal relationships [13]. These methods are generally categorized into supervised and unsupervised learning methods. Supervised learning methods include classification and regression algorithms, such as eXtreme Gradient Boosting (XGBoost) [14]. Sigmoid Kernel Function Support Vector Machine (SKF SVM) [15], and K-nearest Neighbor (KNN) [16]. These methods are applicable to various types of data, particularly demonstrating strong performance in complex multiomics applications. XGBoost is a sophisticated decision tree algorithm that excels in clinical management and disease diagnosis [17]. Compared with KNN, XGBoost has demonstrated superior performance, leading to more accurate and reliable diagnostic outcomes [18]. SVM transforms the original input data into a higher-dimensional feature space, effectively addressing the problems of small size and high significance [15]. The Generalized Linear Model Network (Glmnet) excels in handling high-dimensional data through Elastic Net Regression (EN) regularization, combining L1 (Lasso) and L2 (Ridge regression) penalties for efficient feature selection and overfitting prevention. Ridge regression and Lasso models can be optimized by adjusting the L1 and L2 parameters [19]. These models have demonstrated impressive performance across numerous studies, making them a popular choice for various predictive tasks.

Machine learning methods have been demonstrated to be effective in cancer prognosis analysis, therapeutic target prediction, exoskeleton device development, and drug target identification [20,21,22]. Similarly, ML approaches have yielded good application results in mining biomarkers related to economic traits in livestock and poultry, as well as in genomic breeding research. For example, Li et al. reported that when phenotypes obtained from live measurements, such as breast muscle width, were used as inputs, the EN algorithm yielded the highest correlation in predicting breast muscle weight [23]. Chen et al. measured 17 live indicators, including body weight, body diagonal length, shank circumference, and subcutaneous fat thickness, in a population of yellow-feathered broilers and used Support Vector Regression (SVR) and Artificial Neural Network (ANN) models to predict breast muscle weight and abdominal fat weight. The authors reported that the SVR model performed well in predicting breast muscle traits (R^2^ > 0.95) [24]. Currently, the integration of ML and multiomics research technology has gained increasing attention. Cho et al. used Random Forest (RF) and Adaptive Boosting (AdaBoost) models to screen for breed-specific single-nucleotide polymorphisms (SNPs) in Yeosan Ogye chickens, achieving an identification accuracy of 100% [25]. Similarly, Wei et al. identified 47 breed-specific SNPs in Taihang chickens via population genetics and ML methods [26]. Liu et al. analyzed differentially expressed genes related to backfat thickness in pigs and reported that the AdaBoost model could accurately predict phenotypic distribution [27]. For chickens, more research has focused on predicting carcass phenotypes or breeds via live phenotypes and genomic variations, whereas studies applying multiomics technologies for phenotype prediction are relatively rare.

In this study, we performed transcriptome sequencing on muscle tissues of commercial broilers and local chickens. By applying different ML methods, we selected feature genes and AS events associated with the meat production trait to accurately distinguish trait distributions. Furthermore, we used the Shapley Additive exPlanations (SHAP) method to investigate the interpretability of the model and identify key genes affecting traits, along with their effect sizes.

## 2. Materials and Methods

### 2.1. Animals and Trait

A total of 312 Xianju chickens (XJ, *n* = 165) and commercial broilers (CB, *n* = 147), which were reared at Sishan Farm (Hangzhou, China), were used in this study. They were fed corn-soybean diets containing 11.43 MJ/kg metabolizable energy (ME) and 16% crude protein (CP) for XJ, and 12.13 MJ/kg ME and 18.3% CP for CB. Fresh feed and water were available *ad libitum*. Based on their growth performance, XJ and CB were slaughtered at marketing age. After 12 h of fasting, XJ chickens were slaughtered on day 140, whereas CB chickens were slaughtered on day 42. Body weight was recorded, and the breast muscle was removed and weighed. The breast muscle weight percentage (BrP) was calculated as previously reported. Additionally, breast muscle samples from each individual were collected and stored at −80 °C after being snap frozen in liquid nitrogen.

The XJ chickens were divided into two groups (high group (*n* = 83) and low group (*n* = 82)) on the basis of the distribution of the BrP trait, and the ratio of the population size in each group was approximately 1:1. The same grouping strategy (high group (*n* = 75) and low group (*n* = 72)) was also applied to the CB population.

### 2.2. RNA Extraction, Library Preparation, and Sequencing

Total RNA from muscle samples was isolated using TRIzol reagent (TAKARA, Beijing, China). RNA concentration and integrity were evaluated using an Agilent 4200 Bioanalyzer (Agilent Technologies, Palo Alto, CA, USA) and RNase-free agarose gel electrophoresis. The qualified RNA (RNA Integrity Number > 7) was reverse transcribed into cDNA and randomly fragmented. cDNA was subsequently purified, end-repaired, and poly(A) tails and universal adapters were added. The products underwent size selection and PCR amplification according to the manufacturer’s instructions. Finally, the libraries (paired-end, 150 bp) were sequenced using the NovaSeq 6000 platform (Illumina, San Diego, CA, USA). More than 6 Gb of raw data per individual were generated, totaling more than 2.2 Tb of raw data.

### 2.3. Construction of the Gene Expression Matrix

Low-quality reads were removed using Fastp v0.23.4 software [28], and the remaining reads were accurately aligned to the reference genome (GRCg7b, GCA_016699485.1) using HISAT2 v2.2.1 [29]. Then, file format conversion, sorting, quality control, and indexing were conducted by SAMtools v1.21 [30]. StringTie v2.1.6 [31] was employed for transcript assembly and gene quantification, and an in-house Python script (prepDE.py) was applied to generate a gene count matrix with the parameter: -l 150. Normalization of gene length and sequencing depth was performed to obtain transcripts per kilobase of exon model per million mapped reads (TPM), and the TPM matrix was generated using a modified getTPM.py script.

### 2.4. Identification of Differentially Expressed Genes (DEGs)

DEGs were identified using DESeq2 [32] software (version: 1.34.0) on the basis of the merged gene set (raw count) of each population, and the grouping strategy was as described above. To ensure that potential functional genes were not ignored, |log2FC| > 0.263 and *p* < 0.05 were defined as the significance thresholds.

### 2.5. Identification of Alternative Splicing Events

SUPPA2 v2.3 [33] was used to identify AS events in the muscle tissues of each chicken. AS events were generated from the annotation file of the chicken genome and classified into skipping exon (SE), mutually exclusive exons (MX), alternative 5′ or 3′ splice-site (A5, A3), retained intron (RI), and alternative first or last exon (AF, AL). We subsequently calculated the relative abundance (PSI) value per sample for each transcript on the basis of the gene expression matrix. Finally, we performed differential splicing analysis via a classical method. Thresholds of >0.1 of the absolute value of the PSI difference and <0.05 for the *p* value were regarded as significant. The corresponding transcripts identified as differential AS events were defined as differentially spliced transcripts (DSTs).

### 2.6. Machine Learning Modeling

To screen the crucial genes and AS events affecting breast muscle development, we performed ML analysis on the basis of DEGs and DSTs. From the merged dataset and grouping results, a total of 249 randomly selected samples were selected as the training set, and 63 samples were chosen as the test set. The training set was employed to construct the ML models, and the test set was used to evaluate the predictive power of specific ML models. To identify the best ML models for BrP trait prediction, 10 supervised ML classification models (linear, nonlinear, and integrated ensemble methods) were used, including SKF SVM, ANN, XGBoost, KNN, Glmnet, RF, Decision Tree (DT), Naïve Bayes (NB), Linear Discriminant Analysis (LDA), and Logistic Regression (LR).

The pipeline is as follows: first, a total of 3620 DEGs and 875 DSTs were used for training 10 ML models. The optimal hyperparameters for each model were determined using the grid search approach, and the predictive accuracy was subsequently compared (10-fold cross-validation, 100 repeats). Next, we performed feature selection on the basis of importance scores obtained from the XGBoost and Glmnet models for DEGs and DSTs, respectively. Owing to the differences in the principles of calculating importance scores, we adopted different criteria for feature selection. For the XGBoost model, features were selected when the cumulative importance score reached 95%. For the Glmnet model, features with an importance score greater than 0.2 were selected. We then remodeled the data using the featured DEGs and DSTs and calculated their predictive accuracy. Finally, we evaluated the predictive power of the Recall score, Specificity, Precision, F1 score, Area Under the ROC (Receiver Operating Characteristics) curve (AUC), and Predictive Accuracy using the test set.

$$Recal=\frac{TP}{TP+FP}$$

$$Specificity=\frac{TN}{TN+FP}$$

$$Precision=\frac{TP}{TP+FP}$$

$$F1=2\times \frac{Precision\times Recal}{Precision+Recal}$$

$$Accuracy=\frac{TP+TN}{TP+FP+TN+FN}$$

Here, *TP* represents true positives, *TN* represents true negatives, *FN* indicates false negatives, and *FP* indicates false positives.

The ML modeling and optimal hyperparameter calculations were implemented using the Scikit-learn v1.5.1 [34] in Python v3.8 and the mlr3, mlr3learners, mlr3verse, and mlr3tuning packages [35] in R v4.4. Hyperparameters and evaluating indices (e.g., Recall) for different ML models were calculated on the basis of 100 replications.

### 2.7. Interpretation of ML Models by Shapley Values

To enhance the interpretability of our robust models and obtain a more profound understanding of feature contributions, we employed the SHAP method [36]. Shapley values were calculated through the SHAP package in Python [36], and features were ranked on the basis of the sum of the absolute Shapley values. To visualize ML model interpretation, a beeswarm plot, summary plot, and dependent plot of the top 20 features are displayed. Additionally, a summary plot using the breed variable was generated to evaluate the effects of breed on feature contributions.

### 2.8. Annotation of Feature DEGs and DSTs

Feature DEGs and DSTs were annotated according to the reference genome (GRCg7b) and enriched using Gene Ontology (GO) analysis using KOBAS (http://bioinfo.org/kobasindex.php) (accessed on 30 May 2025) [37]. A *p* value of <0.05 was considered statistically significant. The top GO terms were visualized using the ggplot2 package [38] in R v4.4.

### 2.9. Statistics

To assess and compare the performance of hyperparameter training and different ML models, we conducted a Kruskal-Wallis H test (kruskal.test function) to compare the predictive accuracy of various ML models, and a Mann-Whitney U test (wilcox.text function) was used to assess the improved effect of hyperparameter tuning. All the analyses were conducted in the R environment. A *p* value of <0.05 was regarded as significant.

## 3. Results

### 3.1. Descriptive Summary of the Sequencing Results

**Figure 1:**
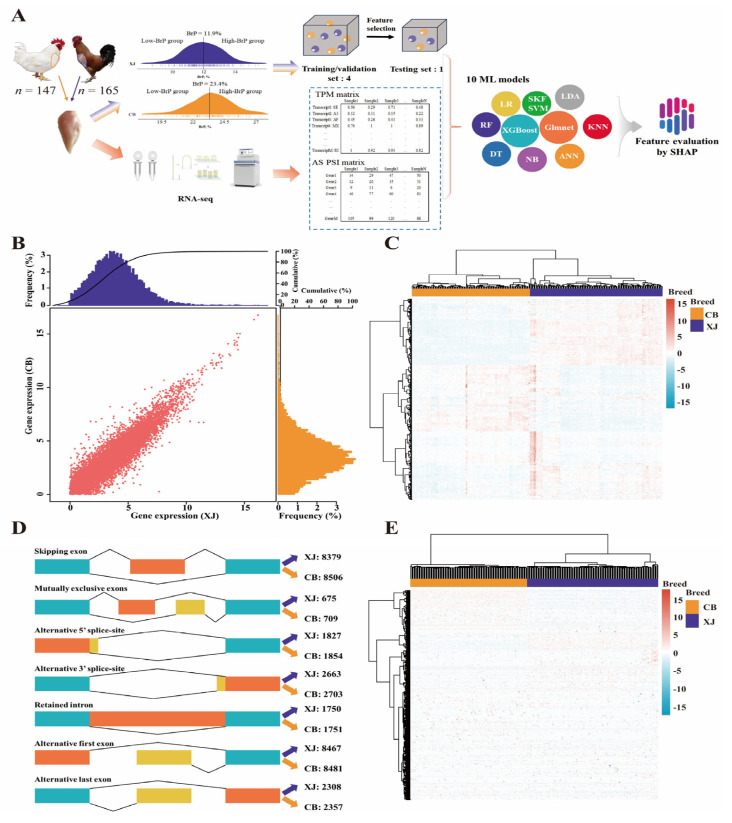
Analytical pipeline and transcriptomic profiling of gene expression and alternative splicing. (**A**) Workflow involving sampling, sequencing, and machine learning analysis. The different balls indicate 10 machine learning models. XJ, Xianju chickens; CB, commercial broilers; ANN, Artificial Neural Network; DT, Decision Tree; Glmnet, Generalized Linear Model Network; KNN, K-nearest Neighbor; LDA, Linear Discriminant Analysis; LR, Logistic Regression; NB, Naïve Bayes; RF, Random Forest; SKF SVM, Sigmoid Kernel Function Support Vector Machine; XGBoost, eXtreme Gradient Boosting. (**B**,**C**) Gene expression profiling of breast muscle tissue in Xianju and commercial broiler chickens. (**D**,**E**) Alternative splicing profiling of breast tissue in Xianju and commercial broiler chickens.

We sampled and sequenced 312 RNA isolates from muscle tissue, including 165 samples from XJ chickens and 147 samples from CB chickens (Figure 1A). A total of 14.8 billion reads (>2225.4 Gb) were produced, more than 95% of which exceeded the quality threshold of Q30 (Supplementary Table S1). After alignment, assembly, and quantification, the expression abundance was standardized using the TPM method, and 11,539 and 11,324 expressed genes (TPM > 1.0) were identified in the XJ and CB populations, respectively. After the two datasets were merged, a global expression profile comprising 11,013 genes was constructed for subsequent analysis (Figure 1B,C and Figure S1). AS events were classified into five types, including SE, A5/A3, MX, RI, and AF/AL events. A total of 71,690 AS events were detected in the reference genome. All the AS events were quantified using the PSI value, and the AS events were retained only when the PSI was greater than 0.1. In total, 2663 A3, 1827 A5, 8467 AF, 2308 AL, 675 MX, 1750 RI, and 8379 SE events in XJ and 2703 A3, 1854 A5, 8481 AF, 2357 AL, 709 MX, 1751 RI, and 8506 SE events in CB populations were defined (Figure 1D). We subsequently corrected these AS events at the transcript level, and 26480 and 26380 transcripts from the XJ and CB populations were compared, respectively (Figure 1E).

### 3.2. Detection of DEGs and DSGs in Each Population

**Figure 2:**
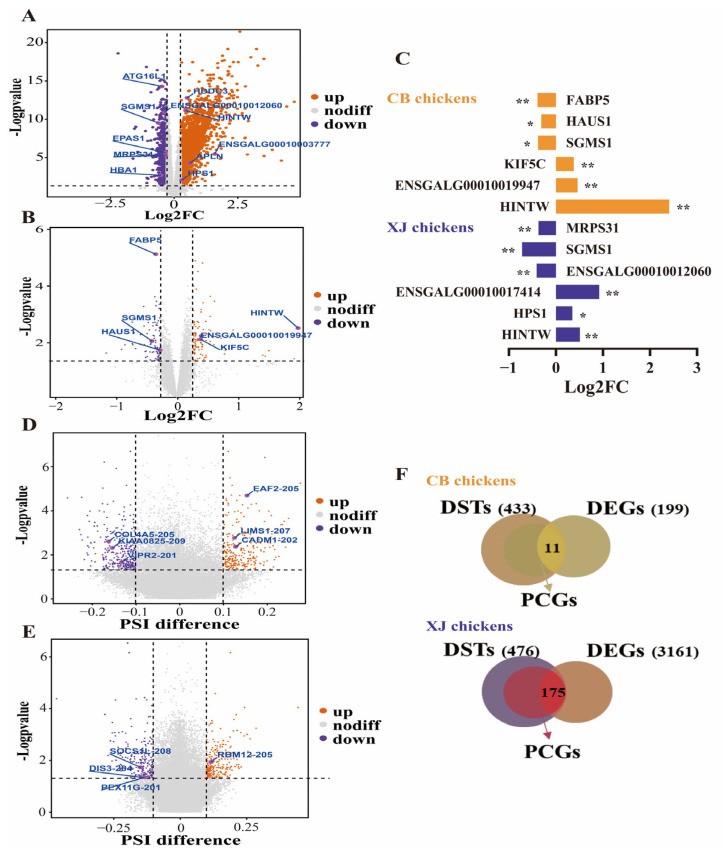
Differential expression and splicing analysis in each chicken breed. (**A**,**B**) Volcano plot for differentially expressed genes in Xianju and commercial broiler chickens. (**C**) RT-PCR results for candidate genes in Xianju and commercial broiler chickens. * indicate *p* < 0.05, ** indicate *p* < 0.01. (**D**,**E**) Volcano plot for differentially spliced genes in Xianju and commercial broiler chickens. (**F**) Venn plot for differentially spliced and expressed genes and protein-coding genes in Xianju and commercial broiler chickens. DEGs, differentially expressed genes; DSTs, differentially spliced transcripts; PCGs, protein-coding genes.

The merged population (*n* = 312) was randomly divided into a training set and a testing set at a ratio of 4:1. To identify the associated genes and AS events, we calculated the DEGs and DSTs in the training set. First, the batch effect was incorporated into the pipeline to identify DEGs between different groups using DESeq2. A total of 3161 and 199 DEGs related to the BrP trait were detected in the XJ and CB populations, respectively (Figure 2A,B, Supplementary Tables S2 and S3). DEGs, including Histidine triad nucleotide binding protein W (*HINTW*), biosis of lysosomal organelles complex 3 subunit 1 (*HPS1*), *ENSGALG00010017414*, etc., were upregulated in the high-BrP group in XJ populations, whereas genes such as *ENSGALG00010012060*, Sphingomyelin synthase 1 (*SGMS1*), and Mitochondrial ribosomal protein S31 (*MRPS31*), were downregulated in the high-BrP group, and these results were proved via RT-PCR (Figure 2C and Figure S2). In CB populations, genes such as *HINTW*, *ENSGALG00010019947*, and Kinesin family member 5C (*KIF5C*) were found to be positively correlated with BrP, whereas *SGMS1*, HAUS augmin-like complex subunit 1 (*HAUS1*), Fatty acid binding protein 5 (*FABP5*), etc., were negatively associated with BrP (Figure 2C).

Next, we integrated the AS events at the transcript level and defined the DSTs by SUPPA2 on the basis of PSI values. A total of 476 and 433 DSTs were identified as significant in different groups in the XJ and CB populations, respectively (Figure 2D,E, Supplementary Tables S4 and S5). In XJ chickens, the DSTs were annotated to 326 nonredundant protein-coding genes, 175 of which were also defined as DEGs (Figure 2F). However, in the CB populations, only 11 DEGs were found among the 295 annotated genes from the 433 DSTs (Figure 2F). However, the significant difference in the number of DEGs between the XJ and CB populations implies that it is difficult to discover candidate genes or AS events by summarizing the results from the two breeds. Therefore, we used all DEGs and DSTs as the inputs for the ML models.

### 3.3. ML Models for BrP

**Figure 3:**
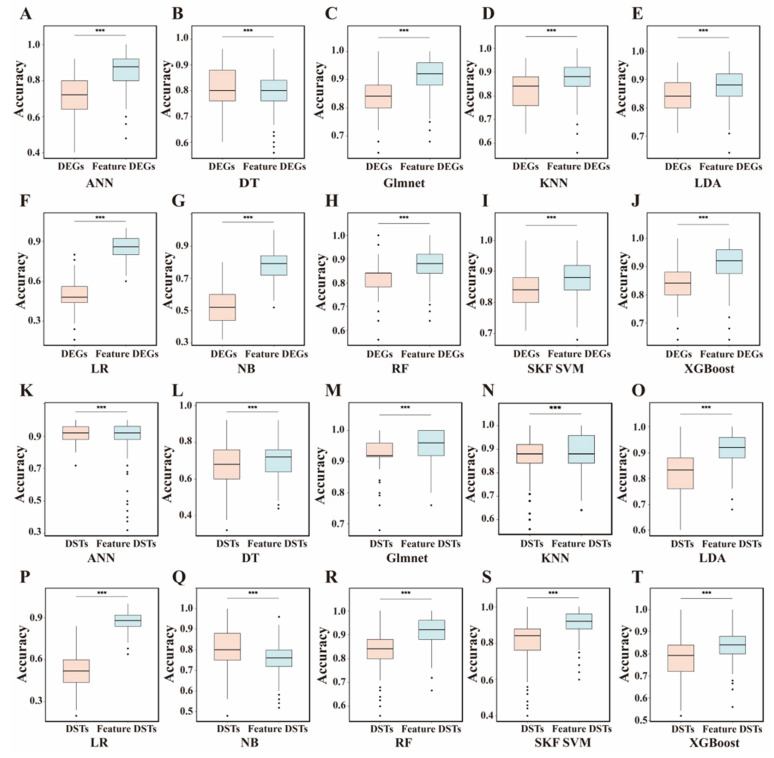
Accuracy of 10 machine learning models based on differentially expressed genes (**A**–**J**) and differentially spliced genes (**K**–**T**). The dot in each plot indicates the individual accuracy in machine learning modeling. ANN, Artificial Neural Network; DEG, Differentially expressed genes; DST, Differentially spliced transcripts; DT, Decision Tree; Glmnet, Generalized Linear Model Network; KNN, K-nearest Neighbor; LDA, Linear Discriminant Analysis; LR, Logistic Regression; NB, Naïve Bayes; RF, Random Forest; SKF SVM, Sigmoid Kernel Function Support Vector Machine; XGBoost, eXtreme Gradient Boosting. *** indicate *p* < 0.001.

To identify molecular markers associated with BrP, we compared the prediction accuracy of ten ML models after hyperparameter tuning using the merged DEG and DST datasets (Supplementary Table S6). For each ML model, a 10-fold cross-validation (CV) method (repeated 100 times) was employed to evaluate the prediction accuracy. For the DEGs, XGBoost yielded the highest accuracy (average accuracy > 0.85, median accuracy > 0.84) in distinguishing between the high- and low-BrP groups, and the Glmnet, SKF SVM, and LDA models also performed well (average accuracy > 0.84, median accuracy > 0.84), whereas the NB and LR methods yielded the lowest accuracy (average and median accuracy < 0.52) (Figure 3A–J). Therefore, we evaluated the feature importance, gain, coverage, and frequency of DEGs with the XGBoost model. A total of 50 feature DEGs (importance score: 0.003~0.25) were selected when the gain reached 95% (Supplementary Table S7). Upon training models using feature DEGs, we observed significant enhancement (*p* < 0.001) in all the ML models (except the DT model) and a superior accuracy score for the XGBoost model (average accuracy > 0.90, median accuracy > 0.92) using the training set (Figure 3A–J). The performance of the Glmnet, SKF SVM, LDA, and RF models (average accuracy > 0.88, median accuracy > 0.88) generally lags behind that of the XGBoost model. The LR model showed the most significant improvement, whereas low accuracy was still observed for the DT and NB models (average accuracy < 0.80, median accuracy < 0.80).

For the DSTs, the prediction accuracy of the XGBoost model was relatively low (average accuracy > 0.77, median accuracy > 0.79), whereas the highest accuracy (average accuracy > 0.93, median accuracy > 0.92) was achieved when the Glmnet model was used (Figure 3K–T). We found that the best values of the alpha and lambda parameters for the Glmnet model were 0 and 0.84, respectively, indicating the use of the ridge regression algorithm in this Glmnet model. The ANN model achieved superior accuracy (average accuracy > 0.90, median accuracy > 0.92) in distinguishing between high- and low-BrP groups. However, the LR (average and median accuracy > 0.52) and DT (average accuracy > 0.67, median accuracy > 0.68) models exhibited poor performance (Figure 3K–T). On the basis of the best lambda for the Glmnet model, we estimated the model coefficient to evaluate the importance of each feature. A total of 95 feature DSTs were selected when the importance score was > 0.2 (Supplementary Table S8). For the training set, the prediction performance of 9 ML models (except for the NB model) significantly improved (*p* < 0.001, Figure 3K–T). The Glmnet model yielded the highest average accuracy of 0.95 (median accuracy > 0.96), and the LDA, ANN, RF, SKF SVM, and KNN models also performed well (average accuracy > 0.89, median accuracy > 0.88). The prediction performance of the LR model improved significantly to 0.87 (average accuracy), although it was still relatively low for the NB and DT models (average and median accuracy < 0.76).

### 3.4. Validation for ML Models Based on Test Dataset

**Figure 4:**
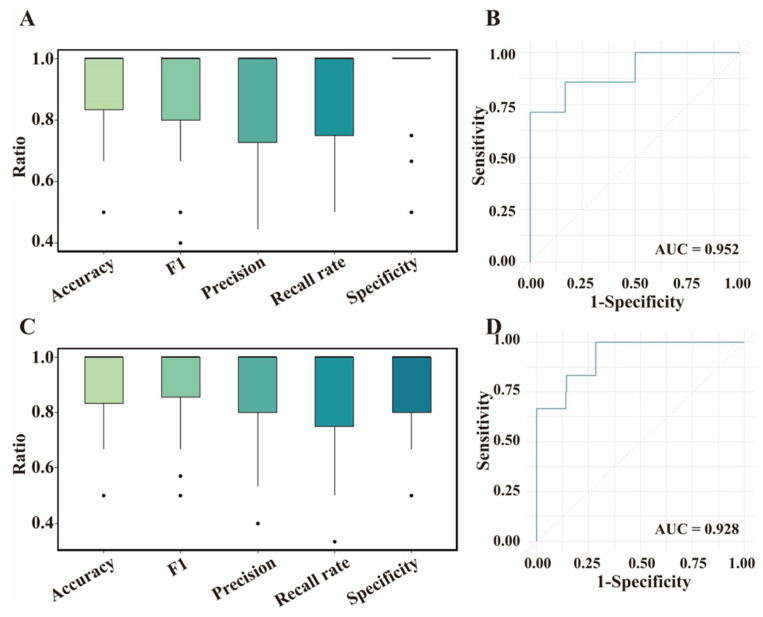
Evaluation of feature differentially expressed genes (DEGs) and differentially spliced transcripts (DSTs) based on different machine learning models in testing set. (**A**) Evaluation of feature DEGs based on XGBoost model. The dot indicates the evaluating parameter of each calculation. (**B**) Receiver operating characteristic (ROC) curve of XGBoost model with feature DEGs. AUC, Area Under the ROC curve. (**C**) Evaluation of feature DSTs based on Glmnet model. (**D**) ROC curve of Glmnet model with feature DSTs.

For the training set, the XGBoost and Glmnet models achieved good predictive performance for the BrP trait on the basis of the DEGs and DSTs, respectively. Next, we used the testing set to validate the generalizability of these models (Figure 4). For feature DEGs, the XGBoost model exhibited outstanding performance in terms of average accuracy (0.89, median accuracy: 1.00), though slightly lower than that with the training set (Figure 4A). Good performance was also observed in terms of average Specificity (0.92, median: 1.00), F1 score (0.88, median: 1.00), Recall rate (0.86, median: 1.00), Precision (0.87, median: 1.00), and AUC (0.95) on the basis of the feature DEGs (Figure 4A,B). We found that the performance of the Glmnet model marginally lagged behind that of the XGBoost model in classifying BrP, with average accuracy, Specificity, F1 score, and AUC of 0.86 (median > 0.83), 0.81 (median: 1.00), 0.87 (median > 0.85), and 0.88 respectively, while the average Recall rate (0.89, median: 1) and Precision (0.88, median > 0.92) performance improved (Figure S3). For feature DSTs, the Glmnet model performed well using the test set, achieving an average accuracy of 0.90 (median: 1.00). However, the average accuracy was slightly lower than that using the training set. The average Recall rate, Specificity, F1 score, Precision, and AUC all reached 0.90 (median: 1.00). However, the XGBoost model performed poorly when predicting the BrP trait using DSTs, and the evaluation metrics (e.g., accuracy, F1 score, and Recall rate) were less than 0.87 (median: 0.80~1.00, Figure S4). Overall, the XGBoost and Glmnet models were suitable for predicting BrP on the basis of the DEGs and DSTs, respectively.

### 3.5. Evaluation of Feature Contributions by Shapley Values

**Figure 5:**
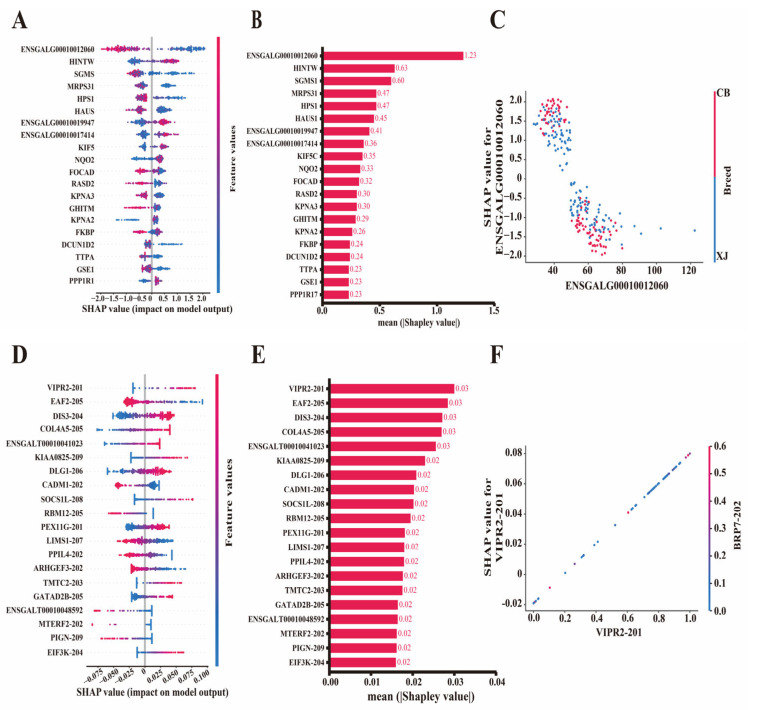
Feature evaluation by SHAP method based on XGBoost and Glmnet models, respectively. (**A**,**B**) Beeswarm and bar plots for top 20 feature differentially expressed genes evaluated via SHAP method based on XGBoost model. (**C**) Dependent plot for *ENSGALG00010012060* based on XGBoost model. (**D**,**E**) Beeswarm and bar plots for top 20 feature differentially spliced transcripts evaluated via SHAP method based on Glmnet model. (**F**) Dependent plot for VIPR2-201 based on Glmnet model. SHAP, Shapley Additive exPlanations.

Although we narrowed the candidate molecular markers related to the BrP trait by feature importance score, no insights were provided into the impact of individual observations or how the direction (positive/negative) of features impacts predictions. Therefore, we computed Shapley values on the basis of DEGs and DSTs using the top-performing XGBoost and Glmnet models. Different ranks of feature contributions were obtained by different model evaluations, and the top 20 influential features with the highest average Shapley values are shown in Figure 5. For DEGs, the Shapley values and summary plot revealed that *ENSGALG00010012060* (1.22) was the most influential gene according to the XGBoost model, and *HINTW* (0.61), *SGMS1* (0.59), *HPS1* (0.5), and others (~0.5) also contributed significantly to BrP prediction (Figure 5A,B, Supplementary Table S9). To better illustrate the contribution of candidate genes to the model’s predictions and the prediction process, we generated dependent plots based on all the samples (Figure S5). *ENSGALG00010012060*, *SGMS1*, and *HPS1* contributed negatively to the model, whereas *HINTW*, *ENSGALG00010019947*, and *ENSGALG00010017414* contributed positively (Figure 5C and Figure S5). Furthermore, we determined that the breed variable had the strongest interaction with *ENSGALG00010012060*, the Shapley value of which was negatively correlated with the expression level of ENSGALG00010012060 (Figure 5C). These crucial genes also had significant effects when the Glmnet model was applied, especially the negative contributor *ENSGALG00010012060* (Figure S6, Supplementary Table S10). Tudor domain containing 3 (*TDRD3*) emerged as the most influential gene in predicting BrP, which also contributed positively to the model. Ras homolog family member B (*RHOB*), Glutamyl-tRNA amidotransferase subunit B (*GATB*), and Protein phosphatase 1 regulatory subunit 17 (*PPP1R17*) were also found to be positive contributors (Figure S7, Supplementary Table S10).

The top 20 influential DST features are shown in Figure 5D. Vasoactive intestinal peptide receptor 2 (*VIPR2*-201, 0.03) was the most influential transcript for predicting BrP (Figure 5E). Other transcripts, such as ELL associated factor 2 (*EAF2*-205, 0.03), DIS3 homolog (*DIS3*-204, 0.03), Collagen type IV alpha 5 chain (*COL4A5*-205, 0.03), and *ENSGALT00010041023* (0.03), had similarly Shapley values to that of *VIPR2*-201 according to the Glmnet model (Figure 5E, Supplementary Table S11). The dependent plots revealed that *VIPR2*-201, *DIS3*-204, and *COL4A5*-205 were major positive contributors, whereas *EAF2*-205, Cell adhesion molecule 1 (*CADM1*-202), and RNA binding motif protein 12 (*RBM12*-205) were negative contributors (Figure 5F and Figure S8). A strong interaction between the top feature *VRIP2*-201 and *RBP7*-202 was also observed (Figure 5F). *EAF2*-205 ranked as the top feature according to the XGBoost model, followed closely by *DIS3*, *COL4A5*, and *ENSGALT00010041023* (Figures S9 and S10, Supplementary Table S12). Therefore, DSTs *VIPR2*-201 and *EAF2*-205 were considered important molecular markers for predicting BrP.

### 3.6. Evaluation of Breed Effect on the Prediction Results

**Figure 6:**
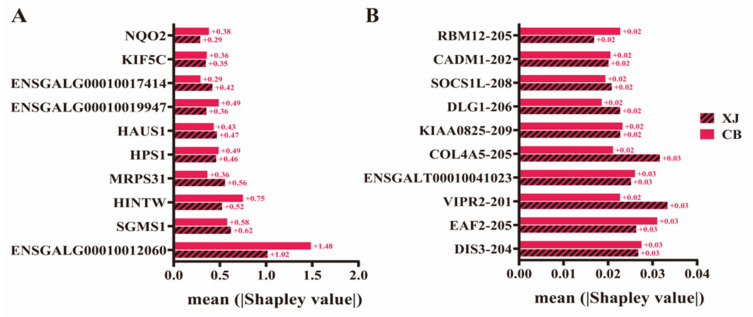
Feature contributions in each chicken breed based on different machine learning models. (**A**) Contributions of differentially expressed genes in each chicken breed based on XGBoost model. (**B**) Contributions of feature differentially spliced transcripts in each chicken breed based on Glmnet model.

To confirm the effect of breed on the prediction results, Shapley values were calculated separately for each chicken breed. For DEGs, *ENSGALG00010012060* contributed more to the prediction results in the CB and XJ populations based on the XGBoost model (Figure 6A, Supplementary Table S13). The genes *HINTW*, *SGMS1*, *MRPS31*, *HAUS1*, *ENSGALG00010019947*, and *HPS1* were identified as key genetic determinants contributing most to the BrP prediction in both chicken breeds (Figure 6A, Supplementary Table S13). While the contribution sizes of specific features were different, the importance rankings of them demonstrated strong concordance in the two breeds. Similar results were obtained with the Glmnet model; *TDRD3* and *C17orf58* were the most important features in BrP prediction in the XJ and CB populations (Figure S11, Supplementary Table S14). For DST features, while *VIPR2*-201 and *COL4A5*-205 showed greater contributions in the XJ population, and *DIS3*-204 and *EAF2*-205 demonstrated higher predictive contribution than *VIPR2*-201 in the CB population, all these genes consistently ranked within the top 5 of feature importance in two breeds according to the Glmnet model (Figure 6B, Supplementary Table S15). Similar to the results according to the Glmnet model, the XGBoost model revealed *EAF2*-205 was significantly influential in both breeds, although it was more important in the XJ population (Figure S12, Supplementary Table S16). The top features –*EAF2*-205, *ENSGALT00010058610*, LIM zinc finger domain containing 1 (*LIMS1*-207), Prolyl 4-hydroxylase subunit alpha 3 (*P4HA3*-202), among others—were identified as the key contributors to BrP prediction in each breed (Figure S12, Supplementary Table S16). In general, breed effects were minimized effectively, having only a small impact on the prediction results, although certain genes exhibited notable breed-specific differences.

### 3.7. Annotation of Feature DEGs and DSTs

**Figure 7:**
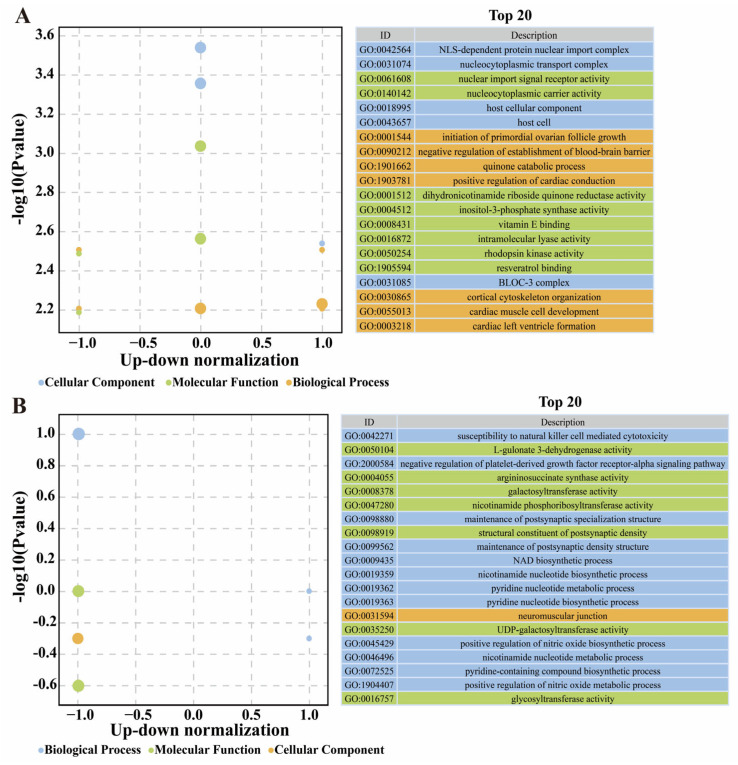
Enrichment of Gene Ontology based on feature genes (**A**) and spliced transcripts (**B**), respectively.

On the basis of the above findings, a total of 50 DEGs and 95 DSTs that could potentially influence BrP classification were identified. These genes and transcripts underwent annotation and GO enrichment. Among the DEGs, the significantly enriched GO terms included nuclear import signal and localization functions (e.g., NLS-dependent protein nuclear import complex), cell signaling (e.g., inositol-3-phosphate synthase activity), muscle functions (e.g., regulation of muscle atrophy), and others (Figure 7A). *CSRP3*, T-box 5 (*TBX5*), and Formin-like 2 (*FMNL2*) are related to the structure and function of actin, Forkhead box O3 (*FOXO3*) is positively related to muscle atrophy, and Fibroblast growth factor receptor 4 (*FGFR4*) is correlated with cell population proliferation and development (Supplementary Table S17). The known gene *HINTW* participates in cytoplasmic and nuclear components. For DSTs, GO enrichment highlighted NAD biosynthetic process, neuromuscular junction, and nicotinamide nucleotide metabolic functions (Figure 7B). The most influential gene, *VIPR2*, was correlated with vasoactive intestinal polypeptide receptor activity, and Discs large MAGUK scaffold protein 1 (*DLG1*), Myomesin 2 (MYOM2), Four and a half LIM domains 3 (*FHL3*), and Microtubule associated monooxygenase, calponin and LIM domain containing 3 (*MICAL3*) were correlated with actin function and muscle development (Supplementary Table S18).

## 4. Discussion

Although gene expression and modifications (e.g., AS) constitute the genetic basis of phenotypic variation in animals, the molecular mechanisms governing these processes remain inadequately characterized. Nonlinear regulatory mechanisms predominantly mediate gene expression-splicing interactions that drive phenotypic outcomes [39]. However, conventional differential expression analyses have limited ability to capture such specific relationships. Current approaches face two challenges: (i) limited statistical power due to small sample sizes in individual studies and (ii) excessive confounding factors in meta-analyses, making them inefficient for identifying key regulatory genes. Although large-scale transcriptomic studies over the past two decades have identified muscle development-associated candidates (e.g., *SOX6* [5] and insulin-like growth factor 2 mRNA binding protein 1 (*IGF2BP1*) [40]) in chickens, distinct genetic architectures and regulatory modalities likely exist across breeds. The approach presented in this study overcomes the resolution constraints of conventional analyses while mitigating sample size limitations through intelligent data integration. The resulting models not only identify conserved regulatory nodes across breeds but also detect population-specific genetic determinants, thereby providing a transformative tool for agricultural genomics and precision breeding initiatives.

As a cornerstone of artificial intelligence, ML methods excel at extracting patterns from complex datasets, enabling automated prediction and decision-making. In agricultural genomics, ML has emerged as a pivotal tool for identifying key genetic determinants of economically important traits [41]. In this study, we found that the XGBoost achieves high prediction accuracy (>0.90) for breast muscle when it is based on DEGs. Unlike traditional models relying on in vivo measurements such as body weight, our transcriptome-based approach requires relatively small datasets while achieving higher accuracy. For example, Li et al. reported that the EN model yielded the highest correlation (R^2^ = 0.77) when phenotypic inputs such as body weight were used, whereas the XGBoost model reached R^2^ = 0.75 [23], Similarly, Zhu et al. extracted features from X-ray images of breast muscle and reported that the XGBoost model achieved a correlation coefficient of 0.80, whereas the SVR model achieved a correlation of 0.84 [42]. AS plays a crucial regulatory role in gene expression, translation, and phenotype formation [43]. Zhao et al. utilized ML and deep learning techniques to predict exon splicing complexity via exon-derived features, achieving moderate levels of prediction [44]. However, there have been no reports to date on predicting macro traits in animals on the basis of AS. Our results indicate that, through Glmnet modeling, transcripts such as *VIPR2*-201 and *EAF2*-205 contribute the most to breast muscle trait prediction, whereas the expression levels of their corresponding genes show minimal phenotypic contributions. These findings suggest that AS events may affect outcomes by modulating posttranslational protein function rather than by altering gene expression [45]. Notably, the AS of the FK506 binding protein 5 (*FKBP5*) gene, along with its expression levels, significantly contributes to predicting the BrP trait, providing insights for further investigation into the molecular mechanisms regulating this event. Typically, quantitative trait predictions rely on regression algorithms. However, this study involved two populations—commercial broilers and indigenous chickens—that exhibit significant phenotypic variation in breast muscle traits. By grouping samples on the basis of high and low phenotypes and utilizing classification algorithms, our results offer valuable guidance for breeding strategies across breeds, indicating that classification algorithms are more suitable for this type of research.

XGBoost, SVM, and RF models have demonstrated good performance in phenotypic prediction; however, owing to their complex processes, they are often referred to as black-box models, making them difficult to interpret [46]. The SHAP method contributes significantly to explaining black box models, allowing for local or global feature and model interpretation [47]. In this study, the SHAP results effectively clearly revealed the contribution of the XGBoost model and various features in predicting breast muscle traits. Notably, genes such as *ENSGALG00010012060* and *HINTW* had the highest Shapley values, indicating that these genes are among the most critical regulators associated with the BrP trait. Previous studies have shown that when egg weight and production traits are predicted using the XGBoost model, the SHAP method identifies key factors affecting egg weight, such as age, wind speed, humidity index, and effective temperature, whereas the main factors influencing egg production are age, feed intake, and effective temperature [48]. Guo et al. utilized the AdaBoost model to apply machine learning techniques on transcriptomic data from muscle tissues in pigs, cattle, and sheep; through SHAP evaluation, they identified genes such as Nanog homeobox (*NANOG*), ADAM metallopeptidase with thrombospondin type 1 motif 8 (ADAMTS8), and LIM homeobox 3 (*LHX3*) as key regulators of muscle development across these three species [49]. Although the samples originated from two breeds, the Shapley value analysis revealed that breed had minimal impact on the relationship between feature genes and alternative splicing. Additionally, the breast samples were not collected at multiple ages within the XJ or CB population; therefore, the effects of age and breed on the prediction performance are consistent. Guo et al.’s study also highlighted the clear advantages of ML methods in integrating cross-species data [49].

This study identified new genes such as *ENSGALG00010012060* and *ENSGALG00010019947*, along with known genes, such as *HINTW*, *SGMS1*, *FOXO3*, and *FGFR4*. Studies indicate that *ENSGALG00010012060* and *HINTW* are located on sex chromosomes, with HINTW being a gene unique to the W chromosome in female birds, associated with the active migration of primordial germ cells (PGCs) and sex differentiation [50]. However, the findings of this study suggest that *HINTW* also has a significant effect on muscle development. With respect to *SGMS1*, Onteru et al. demonstrated that this gene significantly affects the average daily feed intake in pigs [51], which in turn influences growth and development. AS events play crucial regulatory roles in muscle development and contraction functions [52]. In this study, we found that the AS of the *VIPR2* gene significantly contributed to the prediction of breast muscle traits. *VIPR2* is known to be a negative regulator of smooth muscle cell proliferation [53], and our findings also revealed that this gene is negatively correlated with phenotype, which is consistent with previous reports. *EAF2* has been shown to inhibit the transcriptional activity of Smad3, thereby suppressing the TGF-β signaling pathway, thereby suppressing the TGF-β signaling pathway, ultimately preventing G1 cell cycle progression and cell migration [54]. Our results indicate that *EAF2* is negatively correlated with phenotype, potentially influencing myocyte development through pathways such as the TGF-β pathway.

## 5. Conclusions

In summary, this study integrated gene expression and alternative splicing (AS) data from two chicken populations to identify key features—Including *ENSGALG00010012060*, *HINTW*, and *VIPR2*-201—that influence breast muscle traits. Machine learning (ML) and SHAP methods were employed to construct a highly accurate predictive model. These findings represent a shift from traditional gene screening toward artificial intelligence-driven approaches in livestock genomics, with direct implications for improving meat production efficiency through molecular breeding strategies.

## Data Availability

All the sequence data generated in this study have been deposited in the Sequence Read Archive (https://www.ncbi.nlm.nih.gov/sra/) (30 May 2025) with the accession codes PRJNA1230849.

## Supplementary Materials

The following supporting information can be downloaded at: https://www.mdpi.com/article/10.3390/biology14081059/s1, Figure S1: Venn plot for expressed genes in breast muscle tissue from CB and XJ populations; Figure S2: RNA-seq results of candidate DEGs from breast muscle samples in CB and XJ populations; Figure S3: Performance of Glmnet model using feature DEGs in testing set; Figure S4: Performance of XGBoost model using feature DSTs in testing set; Figure S5: Dependent plot of feature DEGs evaluated by SHAP in XGBoost model; Figure S6: Feature evaluation results by SHAP based on Glmnet model; Figure S7: Dependent plot of feature DEGs evaluated by SHAP in Glmnet model; Figure S8: Dependent plot of feature DSTs evaluated by SHAP in Glmnet model; Figure S9: Feature evaluation results by SHAP based on XGBoost model; Figure S10: Dependent plot of feature DEGs evaluated by SHAP in XGBoost model; Figure S11: Shapley values for feature DEGs in each breed; Figure S12: Shapley values for feature DSTs in each breed; Table S1: Summary for RNA-seq of breast muscle samples from two chicken breeds; Table S2: DEGs of high- and low-BrP groups in XJ chickens; Table S3: DEGs of high- and low-BrP groups in CB chickens; Table S4: Identification of DSTs in XJ chickens; Table S5: Identification of DSTs in CB chickens; Table S6: Hyperparameters tuning results in each machine learning model; Table S7: Importance scores for feature DEGs based on XGBoost model; Table S8: Importance scores for feature DSTs based on Glmnet models; Table S9: Shapley values for feature DEGs in XGBoost model; Table S10: Shapley values for feature DEGs in Glmnet model; Table S11: Shapley values for feature DSTs in Glmnet model; Table S12: hapley values for feature DSTs in XGBoost model; Table S13: Shapley values for feature DEGs in each breed based on XGBoost model; Table S14: Shapley values for feature DEGs in each breed based on Glmnet model; Table S15: Shapley values for feature DSTs in each breed based on Glmnet model; Table S16: Shapley values for feature DSTs in each breed based on XGBoost model; Table S17: GO enrichment based on feature DEGs; Table S18: GO enrichment based on feature DSTs.

## Abbreviations

The following abbreviations are used in this manuscript:

ANN	Artificial Neural Network
AS	Alternative Splicing
BrP	Percentage of breast muscle weight
DEG	Differentially expressed genes
DST	Differentially spliced transcripts
DT	Decision Tree
Glmnet	Generalized Linear Model Network
KNN	K-nearest Neighbor
LDA	Linear Discriminant Analysis
LR	Logistic Regression
ML	Machine learning
NB	Naïve Bayes
RF	Random Forest
SHAP	Shapley Additive exPlanations
SKF SVM	Sigmoid Kernel Function Support Vector Machine
XGBoost	eXtreme Gradient Boosting
