# Supplementary figures and images for: Deciphering the Gene Expression and Alternative Splicing Basis of Muscle Development Through Interpretable Machine Learning Models
Source: Biology (Basel). 2025 Aug 15;14(8):1059. doi: 10.3390/biology14081059 (PMC12383657; doi:10.3390/biology14081059)

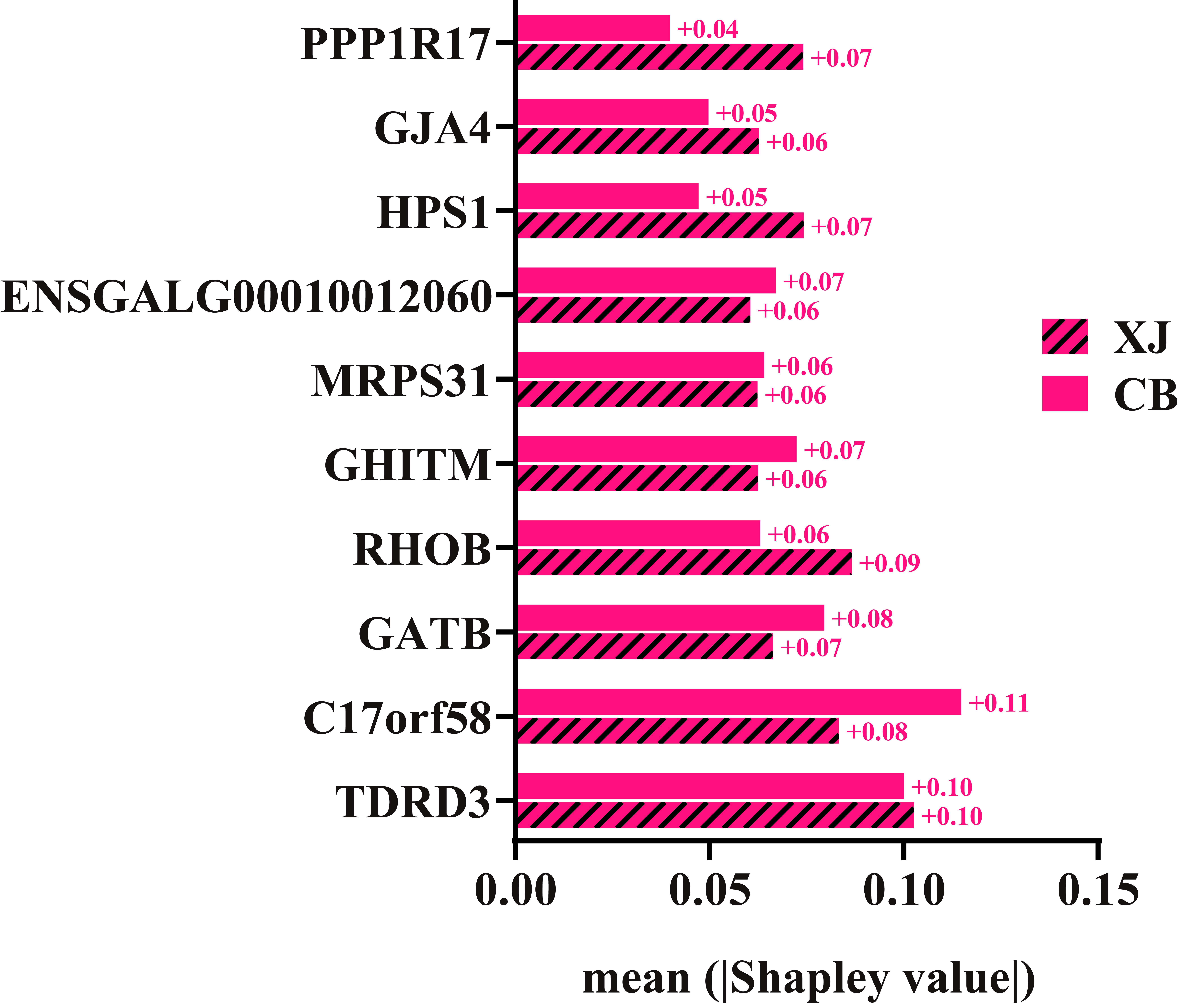

Supplement: Supplementary file 1 [file biology-14-01059-s001.zip › Figure S11.jpg]

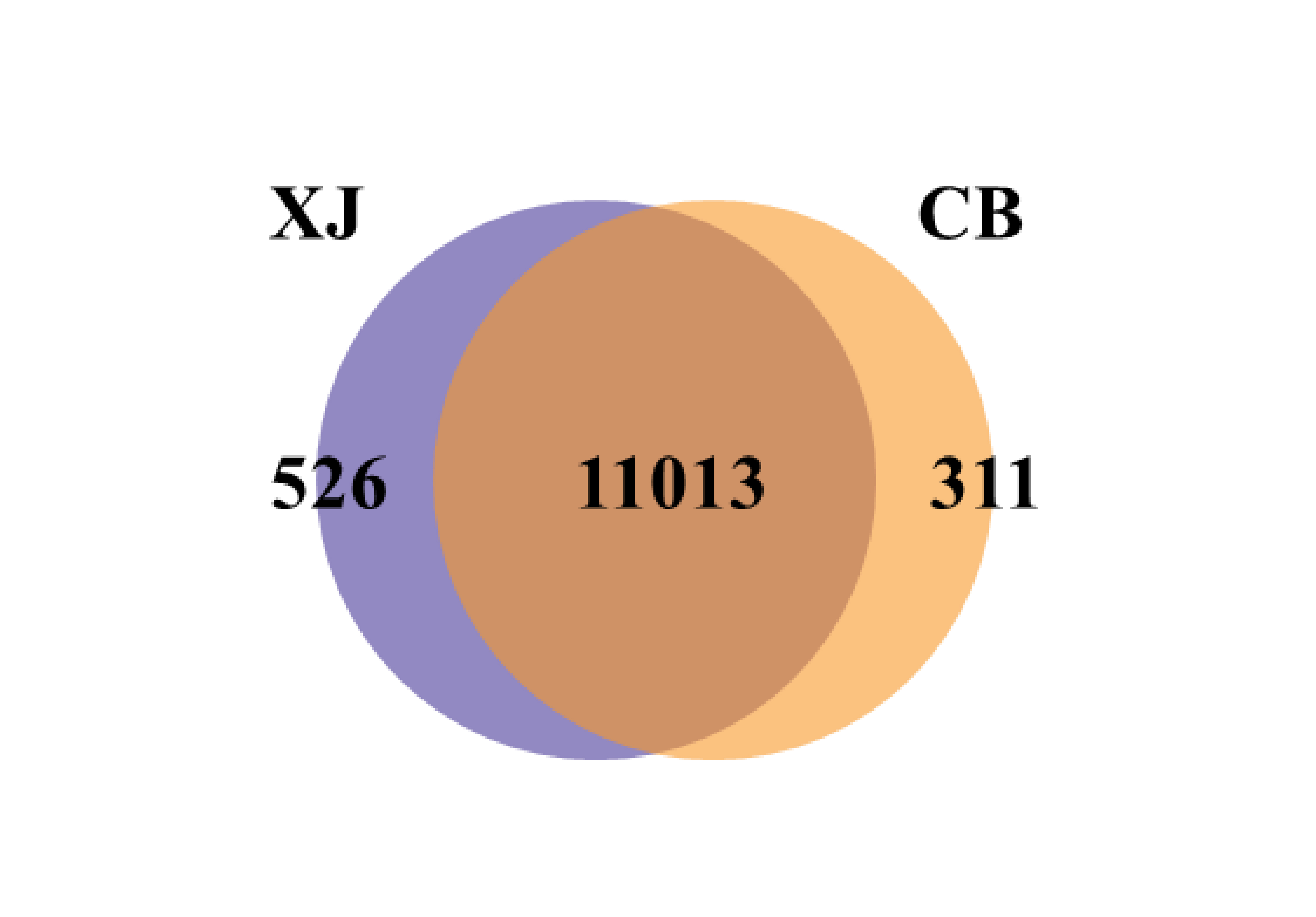

Supplement: Supplementary file 1 [file biology-14-01059-s001.zip › Figure S1.jpg]

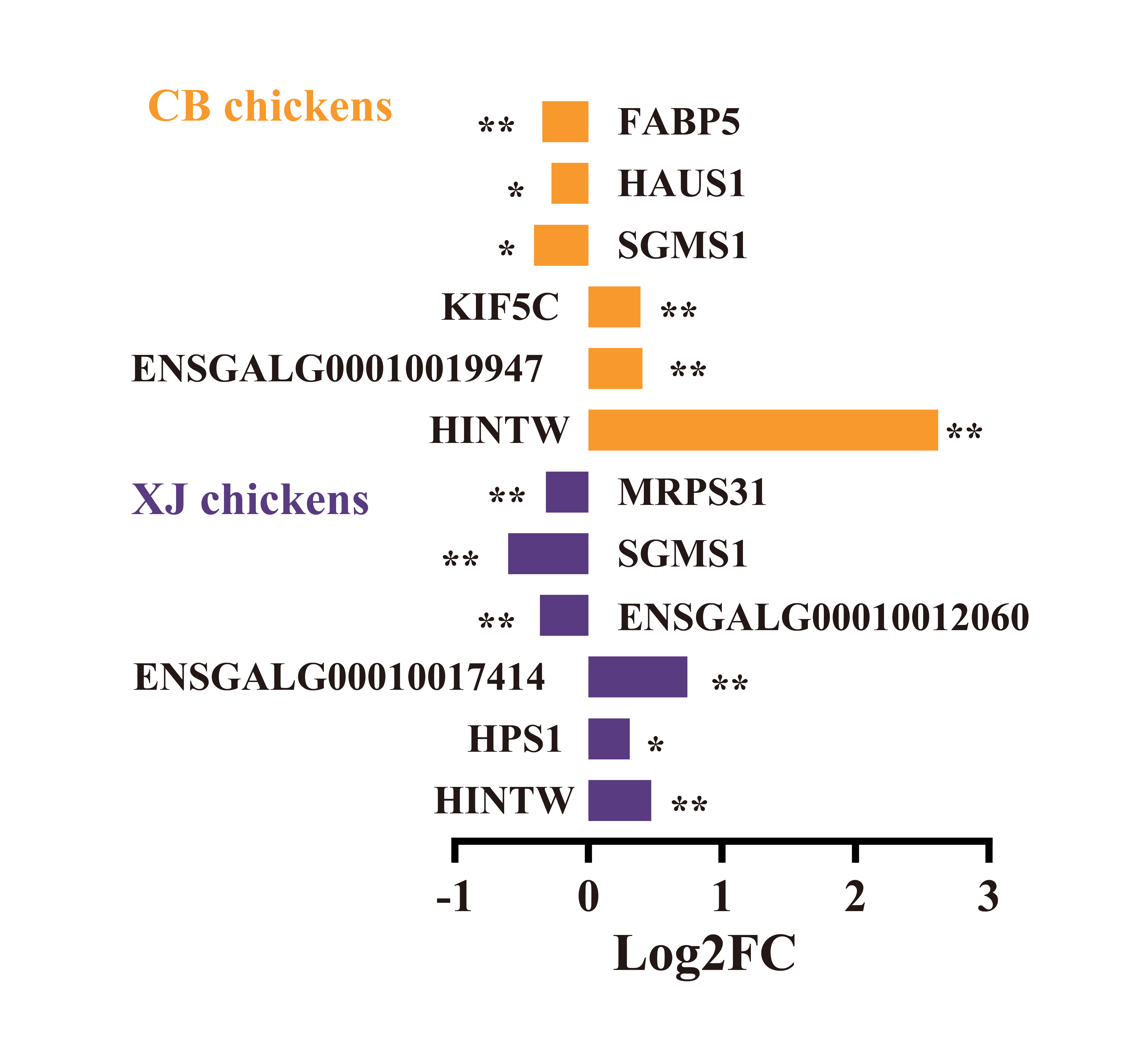

Supplement: Supplementary file 1 [file biology-14-01059-s001.zip › Figure S2.jpg]

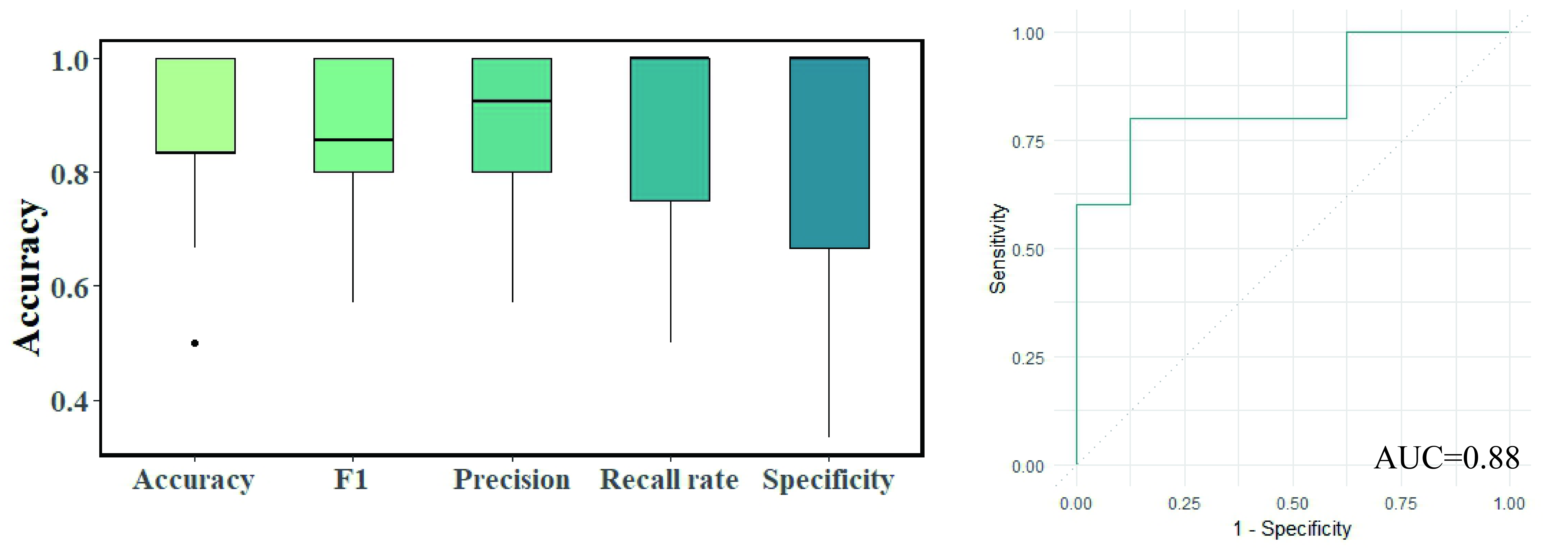

Supplement: Supplementary file 1 [file biology-14-01059-s001.zip › Figure S3.jpg]

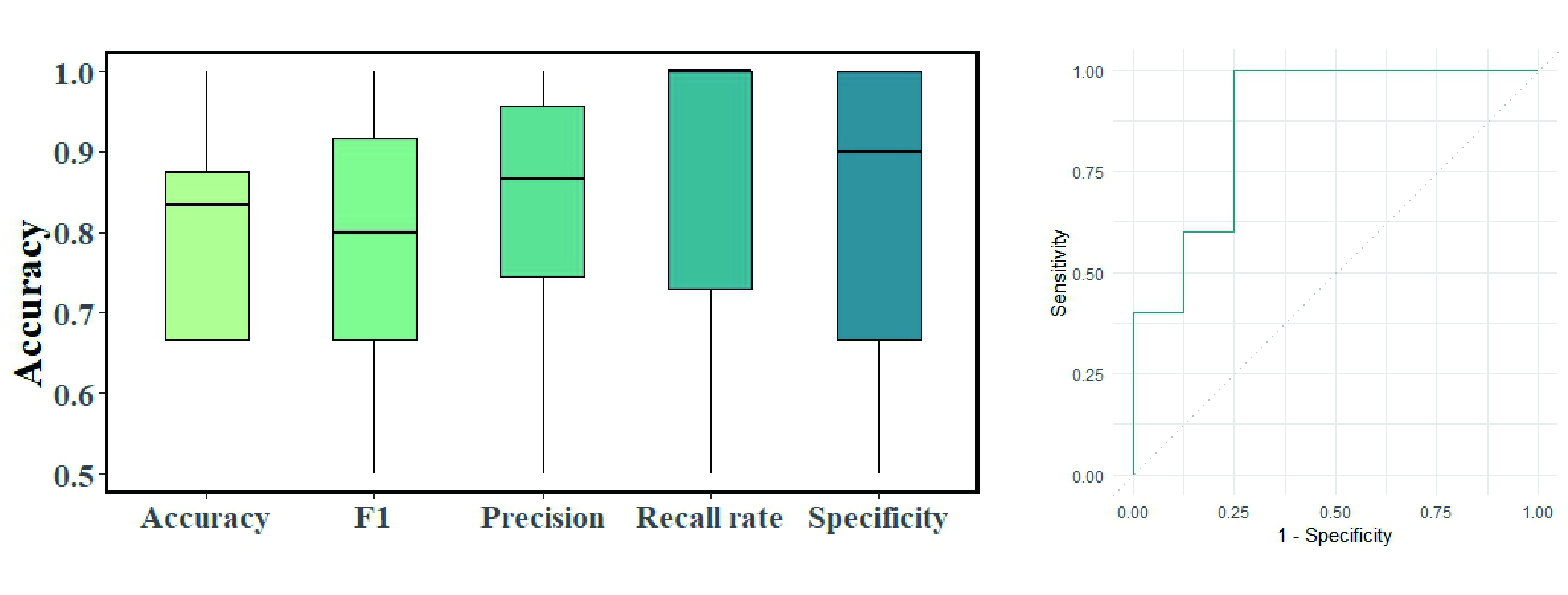

Supplement: Supplementary file 1 [file biology-14-01059-s001.zip › Figure S4.jpg]

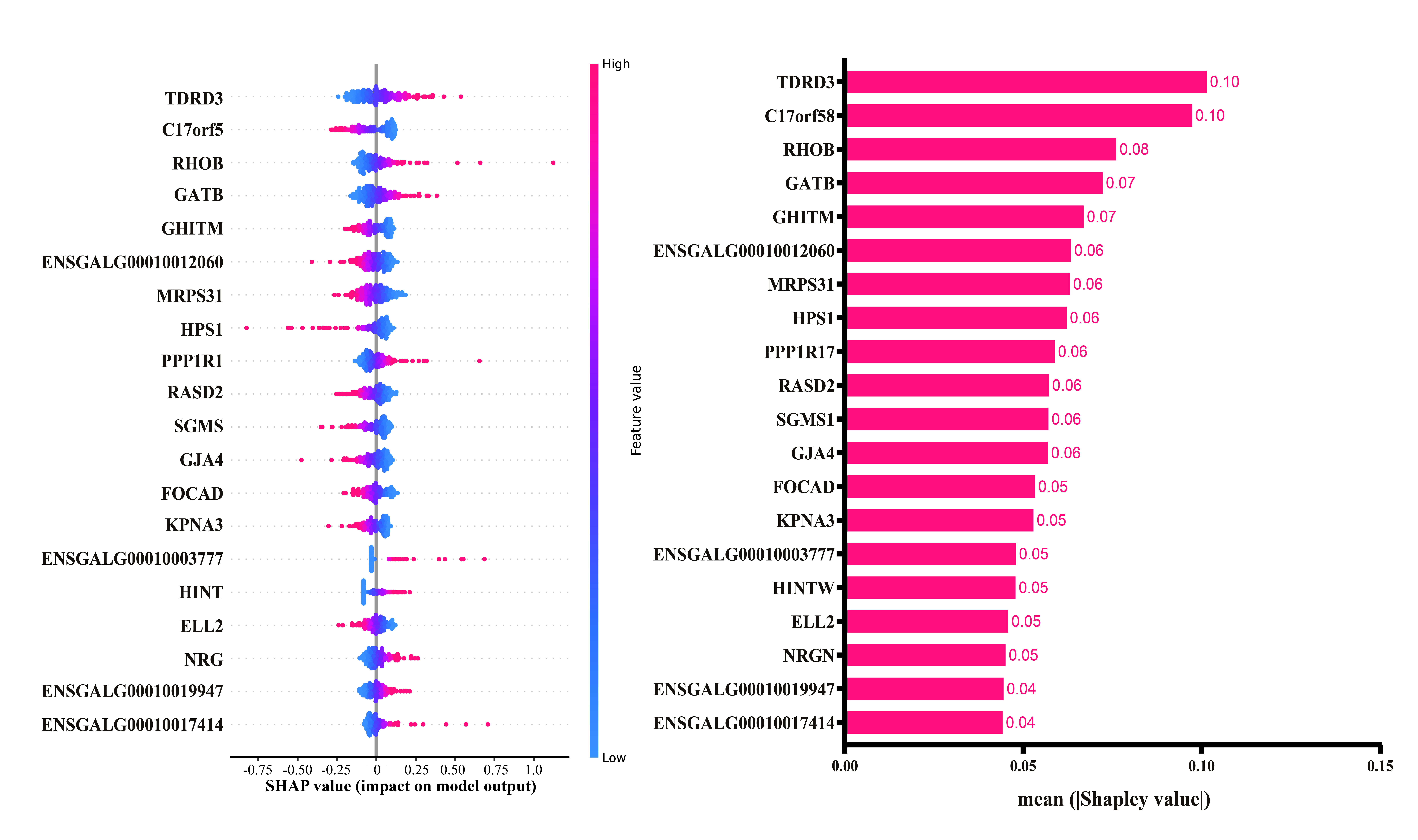

Supplement: Supplementary file 1 [file biology-14-01059-s001.zip › Figure S6.jpg]

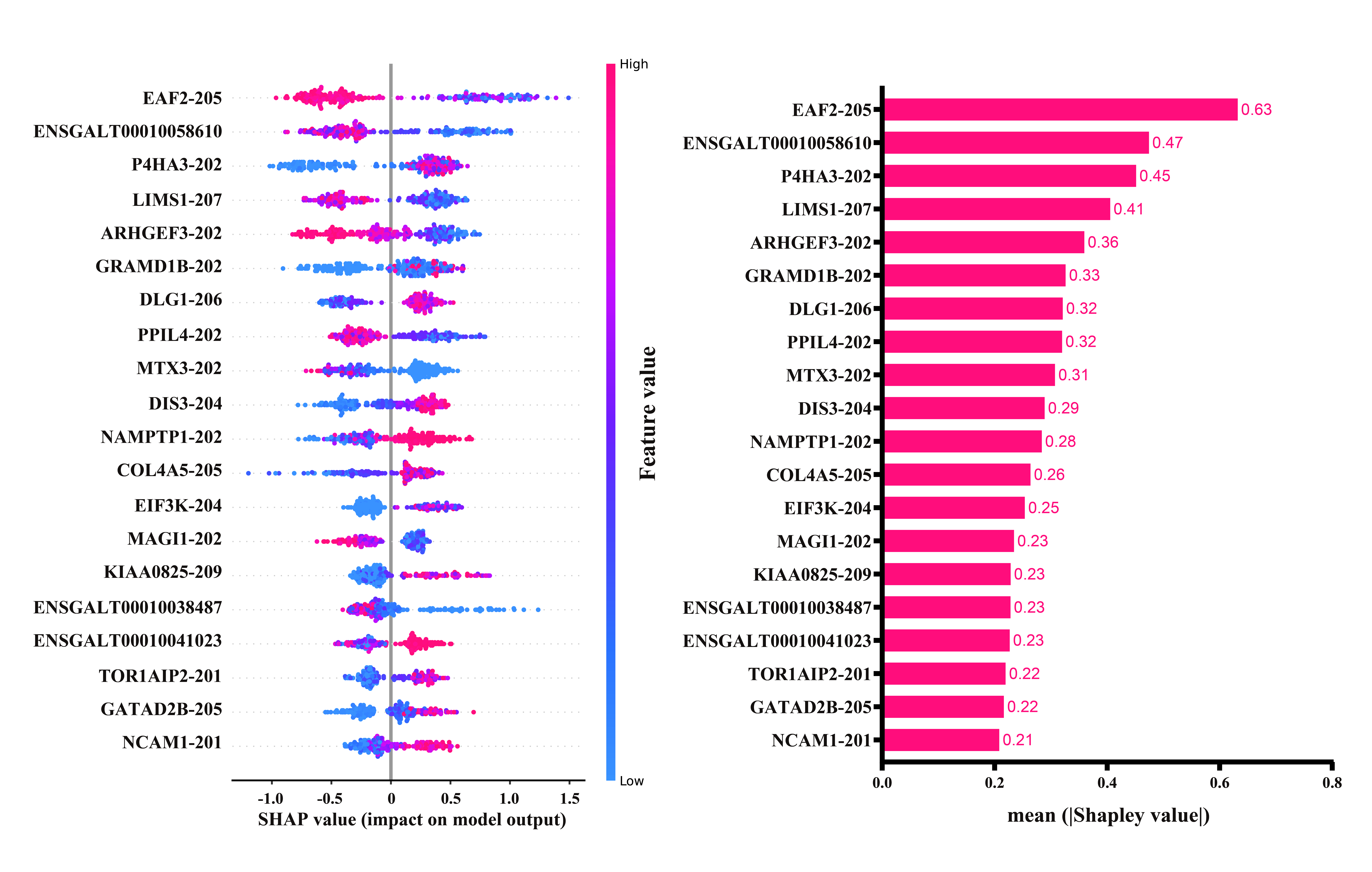

Supplement: Supplementary file 1 [file biology-14-01059-s001.zip › Figure S9.jpg]
